# Development of an Advanced HPLC–MS/MS Method for the Determination of Carotenoids and Fat-Soluble Vitamins in Human Plasma

**DOI:** 10.3390/ijms17101719

**Published:** 2016-10-14

**Authors:** Barbora Hrvolová, Miriam Martínez-Huélamo, Mariel Colmán-Martínez, Sara Hurtado-Barroso, Rosa Maria Lamuela-Raventós, Jiří Kalina

**Affiliations:** 1 Faculty of Science, University of Ostrava, Ostrava 70103, Czech Republic; b.hrvolova@seznam.cz (B.H.); jiri.kalina@osu.cz (J.K.); 2 Department of Nutrition, Food Science and Gastronomy-XARTA-INSA, School of Pharmacy and Food Science, University of Barcelona, Barcelona 08028, Spain; mmartinezh@ub.edu (M.M.-H.); marielcolman@ub.edu (M.C.-M.); sara.hurtado_17@ub.edu (S.H.-B.); 3 CIBER CB06/03 Fisiopatología de la Obesidad y la Nutrición, CIBEROBN, Madrid 28049, Spain

**Keywords:** tandem mass spectrometry, carotenoids, fat-soluble vitamins, human plasma, pilot human study, high antioxidant diet

## Abstract

The concentration of carotenoids and fat-soluble vitamins in human plasma may play a significant role in numerous chronic diseases such as age-related macular degeneration and some types of cancer. Although these compounds are of utmost interest for human health, methods for their simultaneous determination are scarce. A new high pressure liquid chromatography (HPLC)-tandem mass spectrometry (MS/MS) method for the quantification of selected carotenoids and fat-soluble vitamins in human plasma was developed, validated, and then applied in a pilot dietary intervention study with healthy volunteers. In 50 min, 16 analytes were separated with an excellent resolution and suitable MS signal intensity. The proposed HPLC–MS/MS method led to improvements in the limits of detection (LOD) and quantification (LOQ) for all analyzed compounds compared to the most often used HPLC–DAD methods, in some cases being more than 100-fold lower. LOD values were between 0.001 and 0.422 µg/mL and LOQ values ranged from 0.003 to 1.406 µg/mL, according to the analyte. The accuracy, precision, and stability met with the acceptance criteria of the AOAC (Association of Official Analytical Chemists) International. According to these results, the described HPLC-MS/MS method is adequately sensitive, repeatable and suitable for the large-scale analysis of compounds in biological fluids.

## 1. Introduction

A wide range of significant evidence has associated the human diet with chronic diseases in the last decades [1,2,3,4,5]. Based on this evidence, many dietary recommendations and guidelines have been formulated to prevent conditions such as age-related macular degeneration, cardiovascular diseases, some types of cancer, osteoporosis, diabetes, and others [6,7]. Owing to their content of bioactive compounds, notably carotenoids and fat-soluble vitamins, an increment of fruit and vegetable consumption is recommended [8]. Carotenoids, xanthophylls and carotenes are natural fat-soluble, red, yellow and orange pigments characterized by a wide distribution, structural diversity and numerous physio-chemical and biological properties [9]. Particularly due to their antioxidant activity, carotenoids have been the subject of many studies [10,11,12,13,14]. To date, over 700 carotenoid compounds have been identified in various natural sources, and 40 to 50 of these are usually consumed in the human diet [15,16]. Since these antioxidants are synthesized only by plants, algae and fungi [9], their levels in human blood are directly dependent on food intake.

Another group of interesting and useful compounds are fat-soluble vitamins and their metabolites such as retinol, retinol acetate, cholecalciferol, and α-tocotrienol. These compounds also have free radical scavenging properties that allow them to function as antioxidants [17,18]. The presence of fat-soluble vitamins in human tissues and fluids is of vital importance for human health because of their catalytic functions in anabolic and catabolic pathways. Both carotenoids and fat-soluble vitamins can be labeled as fat-soluble micronutrients, and their absorption from the gastrointestinal tract depends on processes responsible for fat absorption or metabolism [19]. An excess or lack of these fat-soluble micronutrients has been associated with the expression of certain diseases. Consequently, there is an increasing demand for the analysis of these antioxidants.

Available methods can determine only a few representatives of the aforementioned fat-soluble micronutrients [20,21,22,23,24,25,26], and few of them can be applied for the simultaneous analysis of compounds in biological samples. Most use high pressure liquid chromatography (HPLC) separation coupled to UV–Vis or diode array detection (DAD) [23,24,25], but with these methods it is extremely challenging to obtain the sensitivity required for the analysis of human fluids, in which the concentration of fat-soluble micronutrients is very low. The problem of sensitivity can be solved by usage of tandem mass spectrometry (MS/MS) detection, although finding general ionization conditions suitable for all targeted analytes is very difficult. A few HPLC–MS/MS methods have been described for the simultaneous analysis of carotenoids and fat-soluble vitamins but usually for no more than 10 analytes [22,25,26,27]. Reported here is a unique HPLC–MS/MS method for the simultaneous determination of 16 carotenoids and fat-soluble vitamins in human plasma.

## 2. Results

### 2.1. HPLC-MS/MS Method Development

#### 2.1.1. Extraction of Carotenoids and Fat-Soluble Vitamins

For the determination of carotenoids and fat-soluble vitamins in a complex matrix such as human plasma, it was necessary to develop an efficient extraction procedure. For our purpose, a double liquid-liquid extraction was designed. The biggest advantage of this extraction procedure is the small amount of human plasma (200 µL) and chemicals required to obtain good quality results. A detailed description is provided in Materials and Methods.

#### 2.1.2. Optimization of Chromatographic and MS/MS Conditions

During the development of the HPLC–MS/MS method, different variations of mobile phase additives were compared: 0.4 g/L ammonium acetate (AMAC), 0.7 g/L AMAC, 1 g/L AMAC, 0.4 g/L AMAC + 0.1% acetic acid (AA), 0.7 g/L AMAC + 0.1% AA, 1 g/L AMAC + 0.1% AA. Table 1 shows the dependency of the MS signal intensity for product ions using multiple reaction monitoring (MRM) in APCI positive mode on the use of LC solvent additives. In general, a combination of AMAC and AA provided better results than the addition of only AMAC to the mobile phases. 0.4 g/L AMAC + 0.1% AA provided the best MS signal intensity for 25-hydroxycholecalciferol, retinol acetate, and cholecalciferol; meanwhile 0.7 g/L AMAC + 0.1% AA provided the best MS signal intensity for most of the analyzed analytes: retinol, cantaxanthin, cryptoxanthin, 13-*Z*-β-carotene, α-carotene, β-carotene, and 9-*Z*-β-carotene. For lutein, the same mass signal intensities were obtained with 0.7 g/L AMAC, 0.4 g/L AMAC + 0.1% AA, 0.7 g/L AMAC + 0.1% AA, and 1 g/L AMAC + 0.1% AA. In the case of *E*-β-apo-8′-carotenal, the MS signals obtained by 1 g/L AMAC, 0.4 g/L AMAC + 0.1% AA, 0.7 g/L AMAC + 0.1% AA, and 1 g/L AMAC + 0.1% AA were also comparable. For α-tocotrienol and astaxanthin, 1 g/L AMAC provided the best MS signal, which was 0.7 g/L AMAC for zeaxanthin, and 0.4 g/L AMAC for 5-*Z*-lycopene. The combination of 0.7 g/L AMAC + 0.1% AA added to both mobile phases was chosen for the final data acquisition because they provided the highest MS signal intensity for product ions of most of the analytes, including the highly problematic carotenes.

**Figure 1:**
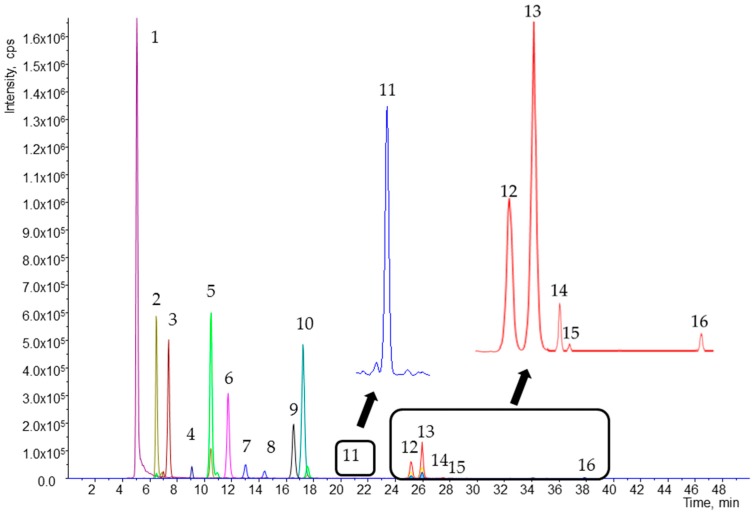
Chromatogram of working standard solutions obtained by high pressure liquid chromatography-tandem mass spectrometry (HPLC-MS/MS) analysis. Peaks: (**1**) retinol; (**2**) 25-hydroxycholecalciferol; (**3**) retinol acetate; (**4**) α-tocotrienol; (**5**) cholecalciferol; (**6**) astaxanthin; (**7**) lutein; (**8**) zeaxanthin; (**9**) cantaxanthin; (**10**) *E*-β-apo-8′-carotenal; (**11**) cryptoxanthin; (**12**) 13-*Z*-β-carotene; (**13**) α-carotene; (**14**) β-carotene; (**15**) 9-*Z*-β-carotene; and (**16**) 5-*Z*-lycopene.

**Figure 2:**
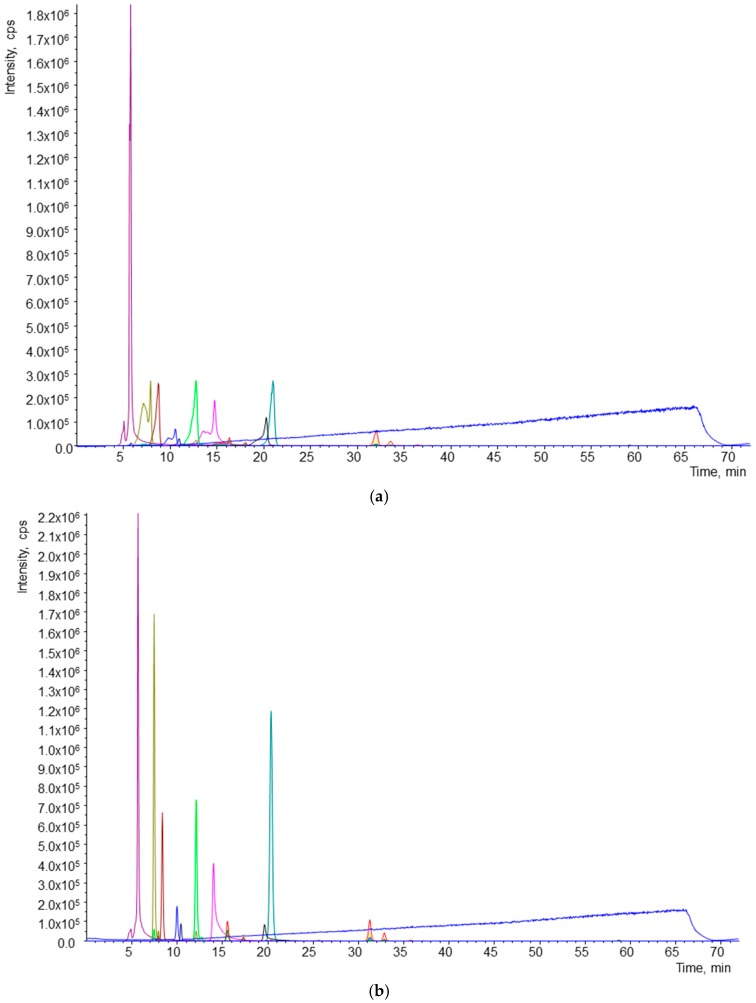
Effect of solvent used for reconstitution of samples. (**a**) Chromatogram of sample reconstituted in methyl *tert*-butyl ether (MTBE); (**b**) chromatogram of sample reconstituted in MeOH.

An excellent separation of 16 analytes in 50 min was accomplished (Figure 1). Flow rate, which allowed good quality resolution, was 0.6 mL/min, and the most suitable injection volume was 20 µL. The resolution and shape of individual peaks were greatly affected by the solvent used for sample reconstitution. During preliminary experiments, methyl *tert*-butyl ether (MTBE) and methanol (MeOH) were used for reconstitution. The effects of the solvents used for reconstitution are shown in Figure 2. Reconstitution by MeOH provided better separation and resolution of all analytes.

Retention time (Rt) and optimal value for mass detection of declustering potential (DP), focusing potential (FP), entrance potential (EP), cell exit potential (CXP), and quantification transitions, with their corresponding collision energy (CE) are shown in Table 2.

### 2.2. Validation of the Method

#### 2.2.1. Limit of Detection (LOD) and Limit of Quantification (LOQ)

LOD and LOQ were determined for individual analytes (Table 3). The sensitivity of the developed HPLC-MS/MS method was evaluated by these two parameters. LOD values were between 0.001 and 0.422 µg/mL, and LOQ values ranged from 0.003 to 1.406 µg/mL according to the analyte. The lowest LOD and LOQ were established for astaxanthin, and the highest for zeaxanthin. The LOD and LOQ values obtained demonstrate that the present HPLC–MS/MS method is suitable for the analysis of carotenoids and fat-soluble vitamins in human plasma.

#### 2.2.2. Linearity

Linearity was evaluated according to signal responses of target analytes from spiked plasma samples at seven different concentrations and by calculating the linear regression. Calibration curves showed linear responses ranging from 0.003 to 10 µg/mL in plasma according to the target analyte. To obtain an adequate accuracy it was necessary to use a weighting factor for most of the analyzed compounds. Sufficient linearity was demonstrated by the coefficient of determination (*r*^2^) values, ranging from 0.990 to 0.999. Linear concentration ranges of individual compounds and associated coefficients of determination are shown in Table 3.

#### 2.2.3. Recovery

The recoveries of analyzed carotenoids and fat-soluble vitamins were between 86.1% and 104.8%. The lowest recovery was achieved in the case of lutein and zeaxanthin. Individual recoveries established during the method validation are presented in Table 3.

#### 2.2.4. Accuracy and Precision

Intra- and inter-day accuracy and precision in three concentration levels with respect to calibration curves were studied. All the analytes met the acceptance criteria by not exceeding 15% RSD. The highest values of RSD% were 14% for 9-*Z*-β-carotene and cryptoxanthin. Obtained accuracy ranged from 85.9% to 114.0%. Values of intra- and inter-day precision are summarized in Table 4 and values of intra- and inter-day accuracy are shown in Table 5. 

#### 2.2.5. Matrix Effect

The final quality parameter of the validation procedure was the matrix effect. Matrix effect values ranged from 86.9% to 108.5% (Table 3). MS signal suppression was achieved for 13-*Z*-β-carotene, cryptoxanthin, lutein, zeaxanthin, β-carotene, 9-*Z*-β-carotene, retinol acetate, 25-hydroxycholecalciferol, cholecalciferol, α-tocotrienol, and 5-*Z*-lycopene. MS signal enhancement was obtained for *E*-β-apo-8′-carotenal, α-carotene, astaxanthin, cantaxanthin, and retinol.

### 2.3. Quantification of Carotenoids and Fat-Soluble Vitamins in Human Plasma

Concentrations of carotenoids and fat-soluble vitamins found in the human plasma after a high antioxidant intervention are shown in Table 6. In plasma samples from eight volunteers the following carotenoids and fat-soluble vitamins were found at quantifiable concentrations: retinol, 25-hydroxycholecalciferol, cantaxanthin, β-carotene, and α-carotene. The highest concentration was obtained in the case of β-carotene (2634.1 ± 1870.3 nM). Retinol acetate, astaxanthin, *E*-β-apo-8′-carotenal, and cryptoxanthin were also detected in plasma but at concentration levels lower than their LOQ. The other targeted analytes were not detected.

## 3. Discussion

### 3.1. HPLC-MS/MS Method Development

#### 3.1.1. Extraction of Carotenoids and Fat-Soluble Vitamins

To keep costs of analytical procedures down, it is necessary to minimize the quantity of plasma used, which entails a lower consumption of chemicals. Two hundred µL of human plasma was used in our experiments. In previously published work, quantities such as 200 µL [28,29], 100 µL [23], 800 µL [30], 1 mL [31], and 300 µL [32] of plasma have been required. Isolation of fat-soluble micronutrients from plasma generally consists of two steps. Deproteinization by the addition of methanol, acetonitrile [31,33] or ethanol [23,29,30,33] to plasma is required as a prior step. Deproteinization is usually followed by extraction with hexane [29], heptane [21] or a combination of hexane with another solvent [34]. Solvents used for extraction are very often enriched with an antioxidant, such as tert-butylated hydroxytoluene or ascorbic acid, to protect carotenoids and fat-soluble vitamins [29,32]. In our study, ethanol for deproteinization and n-hexane enriched with BHT at a concentration of 100 mg/L were chosen. The designed extraction technique is simple, repeatable, non-time consuming and provides good recoveries (Table 3).

#### 3.1.2. Chromatographic and MS/MS Conditions

Numerous HPLC-DAD methods have been designed to determine carotenoids and fat-soluble vitamins [23,24,25]. However, chromatographic techniques coupled to mass detection used for the analysis of a wide range of analytes are scarce. The available HPLC–MS or HPLC–MS/MS methods analyze fewer analytes in comparison with the method described here [25,26,29,32,35,36]. During the development of this method, different combinations of solvents for the preparation of mobile phases and gradients were tested: A = 80:20 MeOH/W (v/v), B = 78:20:2 MTBE/MeOH/W (v/v/v); A = MeOH, B = MTBE and A = MeOH, B = 80:20 MTBE/MeOH (v/v). At the beginning of the study, the total run time for an adequate separation of our analytes was 72 min with the following linear gradient used for B (t (min), %B): (0.0, 90); (40.0, 40); (60.0, 6); (62.0, 90); (72.0, 90) [24]. Before the validation of the method, the resulting total run time was 50 min, with the linear gradient as described in Materials and Methods.

LC solvent additives such as formic acid, acetic acid, propionic acid, ammonium acetate, ammonium formate, and others are usually used in HPLC–MS and HPLC–MS/MS analyses to enhance the mass signal [22,25,26,35,36,37,38]. For example, Kopec et al. [25], who analyzed carotenoids, retinyl esters, α-tocopherol and phylloquinone in chylomicron-rich fractions of human plasma, compared the effects of adding water (no additive), formic acid, and ammonium acetate to the mobile phase, obtaining the best results with the addition of ammonium acetate. Ammonium acetate for mass signal enhancement was also used for the analysis of carotenoids by Meulebroek et al. [26] and Arathi et al. [22]. Another preferred LC solvent additive for MS analysis of carotenoids is ammonium formate [35,36]. In our preliminary experiments, all tested mobile phases were enriched with AMAC at a concentration of 0.4 g/L. Then, the effects of the following additives and their combinations were compared: 0.4 g/L AMAC, 0.7 g/L AMAC, 1 g/L AMAC, 0.4 g/L AMAC + 0.1% AA, 0.7 g/L AMAC + 0.1% AA, 1 g/L AMAC + 0.1% AA (Table 1). Due to providing the best MS signal enhancement for the most problematic analytes, the combination of 0.7 g/L AMAC + 0.1% AA was chosen for the final data acquisition.

These conditions allowed us to efficiently separate 16 analytes in an acceptable time, with very good resolution and suitable sensitivity for the analysis of biological fluids. To the best of our knowledge, a similar HPLC-MS/MS method for the determination of a large number of the afore mentioned analytes has not been previously published.

### 3.2. Method Validation

The proposed method was fully validated based on the criteria of the AOAC International [39]. Since few methods for the simultaneous determination of a wide range of carotenoids and fat-soluble vitamins using HPLC-MS/MS have been published, an effective comparison of our results was limited. As in Kopec et al. [25], lutein, cryptoxanthin, α-carotene, and β-carotene were analyzed and as in Meulebroek et al. [26], lutein, zeaxanthin, α-carotene, and β-carotene. In comparison with Kopec et al. [25], lower LOD and LOQ values were obtained for lutein, but the values were higher for the other analytes. In comparison with Meulebroek et al. [26], comparable LOD and LOQ values for lutein and α-carotene were obtained, but our values for zeaxanthin and β-carotene were higher. The most often used method for the analysis of carotenoids and fat-soluble vitamins is HPLC coupled to DAD [23,24,25]. In comparison with a previous study of our group [24], LOD and LOQ values achieved by the present HPLC-MS/MS method were lower for retinol, astaxanthin, lutein, E-β-Apo-8′-carotenal, 13-Z-β-carotene, α-carotene, and β-carotene, in some cases, more than 100-fold lower. For cryptoxantin, zeaxantin, and 9-Z-β-carotene we obtained comparable LOD and LOQ values. Thus, our method achieved a considerable improvement in LOD and LOQ values in relation to HPLC–DAD methods.

Another very important quality parameter is recovery. Achieved recoveries were in approximate agreement with those published by Lee et al. [23], Colmán-Martínez et al. [24], and Karppi et al. [29]. Recoveries obtained during our experiments were between 86.1% and 104.8%, in comparison with 87% to 105% reported by Lee et al. [23] for α-tocotrienol, retinol, α-carotene, β-carotene, lutein, zeaxanthin, and canthaxanthin. Karppi et al. [29] published recoveries between 86.8% and 103% for retinol, lutein, zeaxanthin, E-β-apo-8′-carotenal, cryptoxanthin, α-carotene, and β-carotene. Recoveries achieved by Colmán-Martínez et al. [24] ranged from 89% to 107% for retinol, astaxanthin, lutein, E-β-apo-8′-carotenal, 13-Z-β-carotene, α-carotene, β-carotene, cryptoxantin, zeaxantin, and 9-Z-β-carotene. The biggest differences in recovery were for lutein and zeaxanthin, which in our case were 86.1% and 86.2%, respectively. The average recovery for these compounds published in the above-mentioned studies is approximately 100%.

For the quality evaluation of our method, we also calculated the matrix effect. It is essential to calculate this parameter for HPLC-MS/MS methods designed to determine compounds in complex matrices such as human plasma, because the matrix can enhance or suppress the acquired MS signal. The matrix effect calculated for each analyte is shown in Table 3. Data to compare the matrix effect was not found.

Perhaps the most valuable parameters for assessing the potential application of the developed method to real samples were intra- and inter-day precision and accuracy, which were determined by injection of plasma extracts spiked at three different concentrations (low, medium, and high) in 5 replications. Intra-day precision ranged from 0.1 to 14.0 RSD% and inter-day from 0.7 to 13.1 RSD%. Values of intra-day accuracy were between 85.9% and 114%. Inter-day accuracy was between 90.3% and 106.6%. These results are summarized in Table 4 and Table 5. Kopec et al. [25] report an accuracy ranging from 93% to 102%, and inter-day precision from 4.5 RSD % to 15 RSD % for lutein, cryptoxanthin, α-carotene and β-carotene. The highest conformity with our analytes (11 common analytes) is found in the study by Colmán-Martínez et al. [24], whose values ranged from 1 to 15 RSD% for intra-day precision; 4.9 to 15.1 RSD% for inter-day precision; and an accuracy of 90.7%–112.2%. In comparison with Colmán-Martínez et al. [24], the method presented here is more precise and the accuracy is comparable. In any case, based on the intra- and inter-day precision and accuracy, our method is repeatable and reproducible and therefore highly suitable for application to real biological samples such as human plasma.

### 3.3. Quantification of Carotenoids and Fat-Soluble Vitamins in Human Plasma

The plasma samples collected after a high antioxidant intervention were analyzed. As the content of carotenoids and fat-soluble vitamins depends on alimentary intake, we could not compare our results (concentrations in plasma before the intervention) with other results such as those published by Colmán-Martínez et al. [24].

## 4. Materials and Methods

### 4.1. Standards, Solvents and Reagents

Carotenoids and fat-soluble vitamin standards: *E*-β-apo-8′-carotenal, α-carotene, 13-*Z*-β-carotene, cryptoxanthin, lutein, zeaxanthin, astaxanthin, cantaxanthin, β-carotene, 9-*Z*-β-carotene, retinol, retinol acetate, 25-hydroxycholecalciferol, cholecalciferol and α-tocotrienol were purchased from Sigma-Aldrich (St. Louis, MO, USA). The standard of 5-*Z*-lycopene was supplied by CaroteNature GmbH (Ostermundigen, Switzerland).

MeOH of LC-MS grade, *n*-hexane, ethanol and MTBE of HPLC grade, synthetic plasma and butylated hydroxytoluene (BHT) were obtained from Sigma-Aldrich. AMAC and AA of HPLC grade were purchased from Panreac Quimica SA (Barcelona, Spain). Ultrapure water (Milli-Q) was generated by a Millipore system (Millipore, Bedford, MA, USA).

#### Preparation of Standard Solutions

All carotenoid and fat-soluble vitamin standards were prepared at a concentration of 1 mg/mL of MTBE by dissolving 1 mg of each standard in 1 mL of MTBE. All standards were processed and manipulated under dim light to protect against light-induced isomerization or possible degradation. Individual working standards were stored in eppendorf tubes and kept in a freezer at −80 °C until analysis. The stock solution used to spike synthetic plasma was prepared by mixing individual working standards at a concentration of 10 µg/mL in MeOH.

### 4.2. UHPLC-MS/MS Method Development

#### 4.2.1. Instrumentation

HPLC-MS/MS was carried out in an Agilent 1100 HPLC system (Agilent Technologies, Hewlett-Packard, Waldbronn, Germany), consisting of a binary pump and an autosampler coupled to a QTRAP4000 (AB Sciex, Foster City, CA, USA) triple quadrupole mass spectrometer with a DAD detector, which was operated in multiple reaction monitoring (MRM) mode. Chromatographic separation was performed on a reversed phase column YMC Carotenoid S-5 μm, 250 × 4.6 mm (Waters, Milford, MA, USA), maintained at 40 °C.

#### 4.2.2. Chromatographic Conditions

For chromatographic separation, the following combination of mobile phases was used. Mobile phase A consisted of MeOH, AMAC at a concentration of 0.7 g/L and 0.1% of acetic acid. Mobile phase B contained MTBE and MeOH (80:20, *v*/*v*), AMAC at a concentration of 0.7 g/L and 0.1% of acetic acid. The following linear gradient of A was used (*t* (min), %A): (0.0, 90); (10.0, 75); (20.0, 50); (25.0, 30); (35.0, 10); (37.0, 6); (39.0, 90); (50.0, 90). Total run time of analysis was 50 min. The mobile phase flow rate was 600 µL/min, and 20 µL of the sample was injected into the UHPLC system.

#### 4.2.3. MS Conditions

A triple quadrupole mass spectrometer QTRAP4000 (Sciex, Foster City, CA, USA) equipped with APCI ionization source and controlled by Analyst v.1.6.2 software (Sciex) was used for direct infusion experiments. During the infusion experiments, the equivalent mixture (50:50, *v*/*v*) of mobile phase containing MeOH:W (80:20, *v*/*v*) + 0.4 g/L AMAC and mobile phase containing MTBE:MeOH:W (78:20:2, *v*/*v*/*v)* + 0.4 g/L AMAC was used. All individual standards were injected at a concentration of 1 µL. An atmospheric-pressure chemical ionization source was working in positive ionization mode. After various optimization experiments, the following parameters were chosen to analyze the final samples: curtain gas, 20 psi; source temperature, 400 °C; ion source gas 1 (GS1), 45 psi; ion source gas 2 (GS2), 0 psi; entrance potential (EP), 10 V; collision cell exit potential (CXP), 15 V. Values of declustering potential (DP) were individual for each standard. During the preliminary experiments, the various combinations of precursor-product ions were obtained. The most selective combination for the analyte with the highest MS signal intensity was chosen for final acquisition data. The resulting data acquisition was performed via MRM mode with a dwell time of 120 ms, with 1412 cycles, and between 10 and 14 data points on the peaks. The product ions were generated by collision-activated dissociation (CAD) applied to selected precursor ions in the mass spectrometer collision cell. The chosen MRM transitions and optimal values of DP and collision energy (CE) for all 17 standards are shown in Table 2.

#### 4.2.4. Quality Parameters

The present method was validated according to the criteria of AOAC International [39]. The quality parameters established for the validation of the method were accuracy, intra- and inter-day precision, recovery, limit of detection (LOD), limit of quantification (LOQ), and linearity. LOD is the lowest quantity of a substance that can be distinguished from the absence of that substance (a blank value) within a stated confidence limit. The LOD is estimated from the mean of the blank, the standard deviation of the blank and the confidence factor. The LOD was estimated from the chromatograms of spiked blank plasma at the lowest analyte concentration tested for a signal-to-noise ratio of 3. Similarly, the LOQ is the lowest concentration not only at which the analyte can be reliably detected but at which predefined goals for precision and accuracy are met. The LOQ is at a higher concentration than LOD. The LOQ was determined for a signal-to-noise ratio of 10.

Another quality parameter, recovery, was established by preparing internal calibration curves (synthetic plasma was spiked before the extraction procedure) and external calibration curves (samples were spiked after extraction procedure). Firstly, the concentration had to be calculated by interpolation of individual analyte areas obtained from the samples spiked after the extraction procedure by samples spiked before the extraction procedure. Further, the dependency of ratio analyte concentration to internal standard concentration on calculated concentration was plotted. Then, a linear regression was applied. The slope multiplied by 100 corresponded to the analyte recovery.

To determine the presence or non-presence of a plasma matrix effect, two calibration curves were prepared. MS/MS areas of known amounts of standards (calibration curve prepared by dissolving of working mixture of standards in MeOH–(A)) were compared with those measured in a blank plasma extract spiked, after extraction, with the same amount of the working mixture of standards (B). The ratio (B/A × 100) was defined as the matrix effect (ME). A value of 100% indicates that there was no matrix effect. There was signal enhancement if the value was higher than 100% and signal suppression if the value was lower than 100%.

Linearity was tested by evaluating signal responses of target analytes from spiked plasma samples at seven different concentrations and by calculating linear regression.

Accuracy expresses the closeness of mean test results obtained by the developed method to the actual concentration of the analyte, and was determined by spiking blank plasma with three known concentrations (low, medium, and high with respect to the calibration curve). The accuracy was expressed as the percentage of the ratio of the mean observed concentration and the known spiked concentration in the plasma matrix. The mean accuracy should be within ±15% of the nominal value. Precision of a method is the closeness of agreement between independent test results obtained from homogenous test material under specified conditions of use. Intra-day precision and inter-day precision were studied. It was decided to use five replicates per three concentration levels: low, medium, and high in a single run or in three different days. The precision of the developed method was evaluated by the %RSD (percentage of relative standard deviation of intra- and inter-day repeatability). The values determined at each concentration level should not exceed 15% of RSD.

### 4.3. Method Application to Real Samples: Human Dietary Intervention Study

The human plasma samples were obtained from healthy, non-smoking male volunteers (8 volunteers), aged between 18 and 32 years. The plasma samples in the pilot human study were collected after two weeks of a high antioxidant dietary intervention consisting of an increased consumption of fruits and vegetables. Blood collection was performed in the morning at 08:00 after fasting. Collected blood samples were immediately centrifuged at 3500 rpm for 15 min at 6 °C. Plasma was separated and samples were stored at −80 °C until analysis. For the determination of carotenoids and fat-soluble vitamins, 200 µL of human plasma was used. The study protocol was approved by the Ethics Committee of Clinical Investigation of the University of Barcelona (Spain) (Institutional Review Board IRB00003099; Approval date: 12 April 2016).

#### Extraction of Carotenoids and Fat-Soluble Vitamins

In preliminary experiments, liquid-liquid extraction as previously described by our group in [30] was used for the isolation of selected carotenoids and fat soluble vitamins. For our purpose, the following double liquid-liquid extraction method was designed. Two hundred microliters of ethanol causing deproteinization and 0.5 mL of *n*-hexane/BHT (100 mg/L) were added to 200 μL of human plasma in an eppendorf tube. This step was followed by a vortexing for 1 min and centrifugation at 2070× *g* for 5 min at 4 °C. The upper nonpolar hexane layer was removed from the two-phase liquid system to another eppendorf tube. The remaining aqueous plasma phase was re-extracted by the second addition of 0.5 mL of *n*-hexane/BHT (100 mg/L) followed by vortexing for 1 min and centrifugation at 2070× *g* for 5 min at 4 °C. The upper nonpolar hexane layer was again removed. Both nonpolar layers were combined in an eppendorf tube and evaporated to dryness by a sample concentrator under nitrogen gas at 25 °C followed by a reconstitution with 100 μL of MeOH. Finally, the samples were stored in glass amber vials with inserts in an ultra-freezer at −80 °C until HPLC-MS/MS analysis. In comparison to a previously published extraction method, we have only proportionally changed volumes of the used solvents and we have also exchanged the solvent for reconstitution of a sample. The same design was used to prepare internal and external calibration curves with purified human plasma. Stock solution of synthetic plasma was prepared by dissolving the purified human plasma in 50 mL of Mili-Q water. For this purpose, mixtures of the above-mentioned individual working standards in methanol at 10, 5, 2.5, 1, 0.5, 0.1, 0.05, 0.01 and 0 µg/mL concentrations have been prepared. For preparation of the internal calibration curve, the following extraction procedure was performed on each concentration level. Two hundred microliters of ethanol was added to 200 μL of human plasma in an eppendorf tube. Then, the plasma sample was spiked by 100 μL of stock solution of individual working standards. This step was followed by the addition of 0.5 mL of *n*-hexane/BHT (100 mg/L) and by a vortexing for 1 min and centrifugation at 2070× *g* for 5 min at 4 °C. The upper nonpolar hexane layer was removed from the two-phase liquid system to another eppendorf tube. The remaining aqueous plasma phase was re-extracted as described above. Both nonpolar layers were combined in an eppendorf tube and evaporated to dryness by a sample concentrator under nitrogen gas at 25 °C, followed by a reconstitution with 100 μL of MeOH. The processed samples were also stored into glass amber vials with inserts in an ultra-freezer at −80 °C until HPLC-MS/MS analysis. For preparation of external calibration curve the exact same extraction procedure was performed on each concentration level, but in this case, removed combined nonpolar phases were spiked by 100 μL of stock solution of individual working standards before evaporating to dryness and reconstitution. All samples were manipulated under dim light during the all procedure steps in order to avoid oxidation and/or isomerization of the bioactive compounds.

## 5. Conclusions

A unique HPLC-MS/MS method for the simultaneous quantification of 16 carotenoids and fat-soluble vitamins in human plasma was designed and fully validated. Good quality values of LOD, LOQ, recovery, linearity, matrix effect, accuracy, and precision were obtained by the proposed method. According to our knowledge, no similar HPLC-MS/MS method for the determination of such a large number of analytes has been previously published. In the future, considering the excellent validation results obtained, this method could be used in various applied clinical studies or investigations.

## Figures and Tables

**Table 1:** Influence of LC solvent additives (0.4 g/L ammonium acetate (AMAC), 0.7 g/L AMAC, 1 g/L AMAC, 0.4 g/L AMAC + 0.1% AA, 0.7 g/L AMAC + 0.1% AA, 1 g/L AMAC + 0.1% AA) on MS signal intensity for product ions using MRM in APCI positive mode. The fair blue represents lower intensities of MS signal than the highest obtained intensity under described conditions. The dark blue represents the highest MS signal intensity.

Analyte	0.4 g/L AMAC	0.7 g/L AMAC	1 g/L AMAC	0.4 g/L AMAC + 0.1% AA	0.7 g/L AMAC + 0.1% AA	1 g/L AMAC + 0.1% AA
retinol	2,200,000	1,400,000	1,700,000	1,600,000	2,400,000	1,800,000
25-hydroxycholecalciferol	1,700,000	1,600,000	1,700,000	1,900,000	1,800,000	1,800,000
retinol acetate	660,000	640,000	600,000	720,000	630,000	700,000
α-tocotrienol	88,000	96,000	110,000	100,000	90,000	98,000
cholecalciferol	730,000	790,000	740,000	860,000	800,000	840,000
astaxanthin	400,000	480,000	530,000	370,000	500,000	500,000
lutein	100,000	140,000	130,000	140,000	140,000	140,000
zeaxanthin	1150	1883	1200	1800	1772	1925
cantaxanthin	86,000	190,000	150,000	170,000	200,000	180,000
*E*-β-apo-8′-carotenal	1,190,000	1,900,000	2,000,000	2,000,000	2,000,000	2,000,000
cryptoxanthin	6000	7950	6483	7500	8000	7317
13-*Z-*β-carotene	108,000	100,000	89,000	107,000	110,000	100,000
α-carotene	40,000	40,000	32,000	40,000	43,000	37,000
β-carotene	5350	5000	4467	5000	5367	5242
9-*Z*-β-carotene	1440	1400	1360	1100	1500	1500
5-*Z*-lycopene	4000	3000	2942	3000	2800	2800

Values represent intensities of MS signal obtained for each analyte under specific conditions.

**Table 2:** Optimized values of declustering potential (DP), entrance potential (EP), cell exit potential (CXP) and retention time (Rt). Quantification transitions of the carotenoids and fat-soluble vitamins with the optimal collision energy (eV).

Analyte	Rt (min)	DP (V)	EP (V)	CXP (V)	Quantification Transition	CE (eV)
retinol	5.00	35	10	15	269 → 181	14
25-hydroxycholecalciferol	6.45	58	10	15	383 → 365	17
retinol acetate	7.34	41	10	15	329 → 269	18
α-tocotrienol	9.03	181	10	15	411 → 165	57
cholecalciferol	10.45	60	10	15	385 → 367	24
astaxanthin	11.69	84	10	15	597 → 147	40
lutein	12.98	102	10	15	551 → 429	26
zeaxanthin	14.40	85	10	15	568 → 476	25
cantaxanthin	16.47	70	10	15	565 → 363	15
*E*-β-apo-8′-carotenal	17.16	70	10	15	417 → 325	14
cryptoxanthin	20.71	94	10	15	553 → 535	20
13-*Z*-β-carotene	25.03	48	10	15	536 → 444	24
α-carotene	25.85	120	10	15	536 → 444	24
β-carotene	27.42	85	10	15	537 → 413	28
9-*Z*-β-carotene	28.13	75	10	15	537 → 413	30
5-*Z*-lycopene	37.65	87	10	15	537 → 413	23

**Table 3:** Limit of detection (LOD), limit of quantification (LOQ), recovery, matrix effect, linearity range and correlation coefficient obtained in samples by the HPLC-MS/MS method.

Analyte	LOD ^a^ (µg/mL)	LOQ ^b^ (µg /mL)	Rec. ^c^ (%)	ME ^d^ (%)	Conc. Range ^e^ (µg /mL)	(*r*^2^) ^f^
retinol	0.002	0.005	102.6 ± 8.9	108.5 ± 9.1	0.005–10	0.990
25-hydroxycholecalciferol	0.003	0.011	92.0 ± 3.2	87.3 ± 1.4	0.011–5	0.995
retinol acetate	0.002	0.008	103.4 ± 1.5	95.1 ± 1.7	0.008–10	0.995
α-tocotrienol	0.113	0.376	99.0 ± 5.3	89.9 ± 3.4	0.376–5	0.992
cholecalciferol	0.005	0.018	102.9 ± 2.9	89.6 ± 1.3	0.018–10	0.998
astaxanthin	0.001	0.003	102.3 ± 2.6	100.1 ± 3.3	0.003–1	0.995
lutein	0.008	0.028	86.1 ± 1.4	91.0 ± 2.0	0.028–10	0.992
zeaxanthin	0.422	1.406	86.2 ± 2.2	86.9 ± 3.2	1.406–10	0.992
cantaxanthin	0.002	0.006	100.1 ± 2.5	103.5 ± 8.0	0.006–1	0.992
*E*-β-apo-8′-carotenal	0.003	0.010	103.6 ± 2.1	100.4 ± 1.4	0.010–10	0.994
cryptoxanthin	0.244	0.812	104.8 ± 3.2	94.0 ± 2.3	0.812–10	0.993
13-*Z-*β-carotene	0.056	0.187	100.6 ± 2.0	87.8 ± 1.0	0.187–10	0.992
α-carotene	0.022	0.073	104.2 ± 5.4	102.8 ± 3.9	0.073–5	0.994
β-carotene	0.041	0.138	101.1 ± 2.2	95.7 ± 5.3	0.138–5	0.999
9-*Z*-β-carotene	0.293	0.975	97.4 ± 6.7	91.5 ± 2.4	0.975–10	0.996
5-*Z*-lycopene	0.189	0.631	104.6 ± 8.2	96.5 ± 3.3	0.631–10	0.998

Values are expressed as means ± standard deviation; LOD ^a^: limit of detection; LOQ ^b^: limit of quantification; Rec. ^c^: recovery; ME ^d^: matrix effect; Conc. range ^e^: concentration range; (*r*^2^) ^f^: correlation coefficient.

**Table 4:** Intra- and inter-day precision obtained in samples by the HPLC-MS/MS method.

Concentration	LOW (*n* = 5)				MEDIUM (*n* = 5)				HIGH (*n* = 5)			
Analyte	Day 1 (RSD%)	Day 2 (RSD%)	Day 3 (RSD%)	Inter-Day (RSD%)	Day 1 (RSD%)	Day 2 (RSD%)	Day 3 (RSD%)	Inter-Day (RSD%)	Day 1 (RSD%)	Day 2 (RSD%)	Day 3 (RSD%)	Inter-Day (RSD%)
retinol	11.5	8.4	3.6	3.8	6.0	6.0	6.7	6.7	9.5	9.2	6.8	3.4
25-hydroxycholecalciferol	10.2	10.0	8.0	1.8	4.4	6.0	11.5	3.3	7.2	8.5	2.5	1.7
retinol acetate	8.6	7.0	5.7	0.7	9.2	6.4	6.8	1.3	8.3	11.1	3.6	0.7
α-tocotrienol	4.7	2.9	3.0	3.5	3.5	8.0	8.8	10.4	9.5	3.8	7.9	3.5
cholecalciferol	12.7	10.0	4.6	1.1	7.4	2.8	9.5	2.0	8.0	9.1	5.7	1.0
astaxanthin	9.9	1.0	7.5	2.7	9.5	4.7	8.8	8.8	6.2	7.0	10.3	4.0
lutein	12.1	9.7	4.5	4.1	5.8	5.2	9.6	2.5	6.2	11.8	5.9	2.0
zeaxanthin	7.3	1.9	2.2	5.5	9.0	9.3	7.5	4.8	8.5	10.8	5.1	0.7
cantaxanthin	11.5	3.3	7.0	6.2	11.1	2.9	9.7	6.2	8.5	11.0	9.9	2.9
*E*-β-apo-8′-carotenal	11.5	5.3	5.1	10.6	8.6	8.0	9.7	13.1	5.3	5.8	2.9	9.4
cryptoxanthin	5.2	7.6	8.2	4.4	10.4	10.1	8.3	1.5	6.9	14.0	4.2	0.3
13-*Z-*β-carotene	10.8	3.1	4.6	3.1	4.4	10.6	10.0	9.1	12.3	13.6	13.1	4.2
α-carotene	5.5	9.8	9.4	5.9	0.7	5.3	10.2	5.0	7.1	12.1	8.1	5.7
β-carotene	8.9	12.2	5.7	2.4	8.1	3.7	2.5	3.4	10.5	2.9	7.8	1.8
9-*Z*-β-carotene	7.0	10.0	5.1	1.3	3.3	4.0	14.0	4.9	8.0	9.7	3.8	4.9
5-*Z*-lycopene	2.8	7.3	4.9	5.2	5.1	0.1	6.5	2.8	13.4	9.2	9.5	5.1

RSD: relative standard deviation; *n*: replicates; *n* = 5.

**Table 5:** Intra- and inter-day accuracy obtained in samples by the HPLC-MS/MS method.

Concentration	LOW (*n* = 5)				MEDIUM (*n* = 5)				HIGH (*n* = 5)			
Analyte	Day 1 (RSD%)	Day 2 (RSD%)	Day 3 (RSD%)	Inter-Day (RSD%)	Day 1 (RSD%)	Day 2 (RSD%)	Day 3 (RSD%)	Inter-Day (RSD%)	Day 1 (RSD%)	Day 2 (RSD%)	Day 3 (RSD%)	Inter-Day (RSD%)
retinol	100.6 ± 11.4	97.7 ± 8.6	105.3 ± 3.6	101.2 ± 3.7	98.8 ± 6.0	104.4 ± 6.0	91.4 ± 6.7	98.2 ± 6.6	100.6 ± 9.5	97.8 ± 9.2	104.6 ± 6.8	101.0 ± 3.4
25-hydroxycholecalciferol	99.2 ± 10.3	97.3 ± 10.1	100.7 ± 8.0	99.1 ± 1.7	101.6 ± 4.5	105.4 ± 5.9	98.8 ± 11.5	101.9 ± 3.3	99.2 ± 7.2	97.3 ± 8.5	100.7 ± 2.5	99.1 ± 1.7
retinol acetate	98.3 ± 8.5	97.1 ± 7.0	97.3 ± 5.6	97.5 ± 0.7	103.3 ± 9.2	106.0 ± 6.5	104.4 ± 7.0	104.6 ± 1.3	98.3 ± 8.3	97.0 ± 11.1	97.9 ± 3.6	97.7 ± 0.7
α-tocotrienol	100.8 ± 4.6	108.0 ± 2.8	104.0 ± 3.0	104.3 ± 3.5	96.4 ± 3.5	86.8 ± 1.7	87.7 ± 3.6	90.3 ± 5.9	100.6 ± 9.5	106.5 ± 3.8	104.0 ± 5.8	103.7 ± 2.9
cholecalciferol	97.9 ± 12.5	95.8 ± 9.8	96.8 ± 4.5	96.8 ± 1.0	103.9 ± 7.3	103.5 ± 11.0	101.6 ± 7.4	103.0 ± 1.2	97.9 ± 8.0	96.0 ± 9.1	97.6 ± 5.7	97.1 ± 1.0
astaxanthin	102.6 ± 9.8	105.3 ± 1.2	99.9 ± 7.5	102.6 ± 2.6	94.9 ± 9.7	92.1 ± 4.7	104.7 ± 5.6	97.2 ± 6.8	102.5 ± 6.2	103.7 ± 7.0	96.3 ± 10.3	100.8 ± 4.0
lutein	94.5 ± 8.2	97.0 ± 9.8	97.7 ± 4.5	96.4 ± 1.7	108.6 ± 5.8	105.2 ± 5.3	103.6 ± 9.5	105.8 ± 2.4	94.7 ± 6.2	97.5 ± 11.8	98.4 ± 5.9	96.9 ± 2.0
zeaxanthin	103.0 ± 7.2	103.8 ± 2.1	112.8 ± 1.7	106.5 ± 5.1	98.2 ± 9.0	97.2 ± 9.3	89.9 ± 7.4	95.1 ± 4.8	100.4 ± 8.5	100.5 ± 10.8	101.7 ± 5.1	100.9 ± 0.7
cantaxanthin	108.9 ± 11.4	104.8 ± 3.2	96.7 ± 7.1	103.5 ± 6.0	94.4 ± 10.9	92.6 ± 2.9	104.0 ± 9.7	97.0 ± 6.3	102.6 ± 8.5	108.0 ± 2.7	98.0 ± 9.9	102.9 ± 4.9
*E*-β-apo-8′-carotenal	111.3 ± 1.3	100.0 ± 5.1	96.6 ± 5.0	102.6 ± 7.5	95.2 ± 4.0	85.9 ± 4.1	103.3 ± 9.4	94.8 ± 9.2	109.0 ± 5.3	114.0 ± 2.3	97.0 ± 2.9	106.7 ± 8.2
cryptoxanthin	101.1 ± 5.2	94.7 ± 7.6	93.0 ± 8.2	96.2 ± 4.4	99.8 ± 10.6	101.6 ± 10.1	102.6 ± 8.4	101.3 ± 1.4	100.1 ± 6.9	99.8 ± 14.0	99.5 ± 4.2	99.8 ± 0.3
13*-Z-*β-carotene	96.5 ± 10.8	102.3 ± 2.9	97.9 ± 4.8	98.9 ± 3.0	108.7 ± 4.3	91.0 ± 10.6	103.5 ± 10.0	101.1 ± 9.0	93.9 ± 12.3	109.0 ± 1.6	97.8 ± 13.1	100.2 ± 7.8
α-carotene	111.5 ± 0.6	105.0 ± 9.5	103.3 ± 9.2	106.6 ± 4.1	86.1 ± 0.7	93.9 ± 5.4	94.2 ± 10.3	91.4 ± 5.0	109.5 ± 2.6	104.1 ± 12.2	102.9 ± 8.5	105.3 ± 3.1
β-carotene	100.3 ± 9.0	96.8 ± 12.0	101.8 ± 5.7	99.6 ± 2.6	99.8 ± 7.9	102.6 ± 3.5	95.9 ± 2.5	99.4 ± 3.4	100.1 ± 10.5	97.6 ± 2.9	101.1 ± 7.7	99.6 ± 1.8
9-*Z*-β-carotene	101.7 ± 7.0	99.2 ± 10.0	100.6 ± 5.1	100.5 ± 1.3	98.9 ± 3.0	107.2 ± 3.9	98.1 ± 14.3	101.4 ± 5.0	100.4 ± 8.0	92.1 ± 9.7	100.6 ± 3.8	97.7 ± 4.9
5-*Z*-lycopene	97.3 ± 2.8	100.7 ± 7.3	90.7 ± 4.9	96.2 ± 5.2	103.5 ± 5.3	98.6 ± 0.1	103.5 ± 6.4	101.8 ± 2.8	96.8 ± 13.4	106.8 ± 9.2	99.5 ± 9.5	101.0 ± 5.1

Values are expressed as means ± relative standard deviation; *n*: replicates; *n* = 5.

**Table 6:** Carotenoids and fat-soluble vitamins quantified in plasma by the HPLC-MS/MS method.

Analyte	Quantification Transition	In Plasma (nM)
retinol	269 → 181	115.2 ± 10.5
25-hydroxycholecalciferol	383 → 365	189.7 ± 32.5
retinol acetate	329 → 269	<LOQ ^a^
α-tocotrienol	411 → 165	n.d. ^b^
cholecalciferol	385 → 367	n.d. ^b^
astaxanthin	597 → 147	<LOQ ^a^
lutein	551 → 429	260.2 ± 138.9
zeaxanthin	568 → 476	n.d. ^b^
cantaxanthin	565 → 363	28.32 ± 12.4
*E*-β-apo-8′-carotenal	417 → 325	<LOQ ^a^
cryptoxanthin	553 → 535	<LOQ ^a^
13-*Z-*β-carotene	536 → 444	n.d. ^b^
α-carotene	536 → 444	100.6 ± 18.6
β-carotene	537 → 413	2634.1 ± 1870.3
9-*Z-*β-carotene	537 → 413	n.d. ^b^
5-*Z*-lycopene	537 → 413	n.d. ^b^

Values are expressed as means ± standard deviation (*n* = 8); <LOQ ^a^: under limit of quantification; n.d. ^b^: not detected.

## Abbreviations

MeOH	Methanol
MTBE	Methyl-*tert*-butyl ether
BHT	Butylated hydroxy toluene
AMAC	Ammonium acetate
AA	Acetic acid
W	Water
CAD	Collision-activated dissociation
DP	Declustering potential
APCI	Atmospheric pressure chemical
EP	Entrance potential
CE	Collision energy
HPLC-MS/MS	High performance liquid chromatography coupled to mass spectrometry in tandem mode
LOD	Limit of detection
LOQ	Limit of quantification
MRM	Multiple reaction monitoring
ME	Matrix effect
MS	Mass spectrometry
CXP	Cell exit potential
RSD	Relative standard deviation
RT	Retention time
